# A proteomic approach to obesity and type 2 diabetes

**DOI:** 10.1111/jcmm.12600

**Published:** 2015-05-09

**Authors:** Elena López-Villar, Gabriel Á Martos-Moreno, Julie A Chowen, Shigeru Okada, John J Kopchick, Jesús Argente

**Affiliations:** a Departments of Endocrinology and Pediatrics, Hospital Infantil Universitario Niño Jesús, Universidad Autónoma de MadridMadrid, Spain; b Oncohematology and Pediatrics, Hospital Infantil Universitario Niño JesúsMadrid, Spain; c Instituto de Investigación La PrincesaMadrid, Spain; d Centro de Investigación Biomédica en Red de Fisiopatología de la Obesidad y Nutrición (CIBEROBN), Instituto de Salud Carlos IIIMadrid, Spain; e Edison Biotechnology Institute, Ohio University, Konneker Research LaboratoriesAthens, OH, USA; f Department of Pediatrics, Heritage College of Osteopathic Medicine, Ohio UniversityAthens, OH, USA; g Molecular and Cellular Biology Program, Ohio UniversityAthens, OH, USA; h Department of Biomedical Sciences, Heritage College of Osteopathic Medicine, Ohio UniversityAthens, OH, USA

**Keywords:** diabetes mellitus type 2, obesity, proteomics, biomarkers

## Abstract

The incidence of obesity and type diabetes 2 has increased dramatically resulting in an increased interest in its biomedical relevance. However, the mechanisms that trigger the development of diabetes type 2 in obese patients remain largely unknown. Scientific, clinical and pharmaceutical communities are dedicating vast resources to unravel this issue by applying different omics tools. During the last decade, the advances in proteomic approaches and the Human Proteome Organization have opened and are opening a new door that may be helpful in the identification of patients at risk and to improve current therapies. Here, we briefly review some of the advances in our understanding of type 2 diabetes that have occurred through the application of proteomics. We also review, in detail, the current improvements in proteomic methodologies and new strategies that could be employed to further advance our understanding of this pathology. By applying these new proteomic advances, novel therapeutic and/or diagnostic protein targets will be discovered in the obesity/Type 2 diabetes area.

- Introduction
- Sample preparation
- Special issues concerning tissue/blood sampling
  Serum samples
- Proteomic methodologies
- Analysis of protein phosphorylation
  Sequential elution of (IMAC) followed by TiO2
  Isobaric tag for relative and absolute quantitation
  Selected reaction monitoring or multiple reaction monitoring
  Label-free quantification
  Electrospray ionization and tandem mass spectrometry MS-n (Nano-ESI-MSn)

- Identification of proteins
- Current and relevant studies of DM and obesity using proteomic approaches
- Concluding remarks

## Introduction

The incidence of diabetes mellitus type 2 (DM2) is increasing at an alarming rate world-wide. This is due, in part, to the dramatic rise in the obesity epidemic as DM2 is a comorbidity frequently seen in obese patients 1. Because not all obese patients develop DM2 and not all patients suffering from DM2 are obese or overweight 2, it is of interest to understand the mechanisms underlying the association between these two entities to predict which patients are at higher risk for developing this disease, and possibly for the development of preventative therapies. DM2 appears as a result of insulin resistance. During the ontogeny of this disease, the pancreas begins to produce greater amounts of insulin to maintain euglycemia that collectively overcomes this resistance. But as time advances, sufficient insulin levels cannot be maintained to control normal levels of glycemia. The clinical characteristics of DM2 appear several years after the onset of this process during which time the person is asymptomatic. Once DM2 clearly appears, it must be attended immediately as it is very difficult and practically impossible to reverse the disease progression. In any event, factors such as exercise and diet can ‘help’, as it is of utmost importance to properly control DM2 through life style, and early intervention of the factors previously mentioned may result in normal glucose levels. Thus, early detection, or even more importantly, the possibility of avoiding DM2 onset would be of extreme benefit.

The alteration of protein structure, function, production and interactions, in part if not completely, contributes to the underlying mechanism of many diseases including diabetes 3. Comprehension of pathological-mechanisms whereby molecular and environmental actions lead to the development and progress of diabetes is important for the prevention and treatment of this disease. In such an endeavour, approaches such as genomics, metabolomics and proteomics are being applied to identify more specific biomarkers for DM2 for its early detection, management and for devising new therapies. In this review, we will focus on the use of proteomics for the identification of novel biomarkers, and perhaps, therapeutic targets for the identification and subsequent treatment of DM2. As in any research study, *efficient and up-to-date methods must be established and used for selection and proper handling of the clinical samples and data* 4.

Simply defined, proteomics is the large-scale study of proteins, particularly of their structures and functions 5,6. Proteins are vital parts of living organisms, as they are the main components of all cellular processes. The term *proteomics* was first coined in 1997 7 as an analogy with *genomics*, the study of an organism’s genome. The word *proteome* is a blend of protein and genome and was coined by Marc Wilkins in 1994 while working on the concept as a PhD student 8. The proteome is the entire set of proteins 8 produced or modified by an organism or system. This will vary with time as a result of distinct requirements or stresses that a cell or organism encounters. Proteomics allows the study of the entire set of proteins, produced or modified by an organism or system and it varies with time and a variety of environmental factors. Indeed, it is formed on the basis of research and development of the Human Genome Project 9,10. Thus, while the genome is static the proteome is dynamic. It is also a fundamental component of functional genomics. While *proteomics* generally refers to the large-scale experimental analysis of proteins in a given cell/tissue/organism, techniques used in the studies can also be applied to protein identification and purification. One of the key methodologies used in this area is mass spectrometry 11. We will discuss in detail the sample preparation step as a key issue for clinical proteomic research in the following section.

## Sample preparation

The sample preparation of body fluids (blood, serum, urine and salivary) or tissues (*e.g*. adipose) is an extremely important pre-requisite for achieving robust and reproducible data *via* proteomics. Once the body fluids and/or tissues have been obtained for future proteomic analyses, they must be frozen rapidly (*i.e*. at −80°C in a bio-banking system) usually in aliquots distributed in several vials to avoid sample deterioration from repeated freeze–thaw cycles. When working with adipose tissue samples, the selection of the representative areas from the human body is extremely important according to the clinical research goal. Tissue handling is also of paramount importance as different tissues will require different homogenization and processing protocols. Several examples are explained in the following sections and in Table1. For clinical proteomic purposes, changes can take place in proteome assays that can easily modify experimental results. For example, contamination of protein-samples can cause results to be skewed, as artefacts and/or contaminants can mask low expressed proteins during mass spectrometry (MS) analysis, and they can also give rise to poor protein-separation during electrophoresis and chromatography, and in addition, artefacts can interact with the given protein, thus changing the 3D-structure 12.

**Table 1:** Research examples of diabetes and obesity studies using proteomic tools

Tool	Sample	Resulting data	Ref.
2DE silver MALDI-TOF	Plasma proteins/blood-sera in ob/ob mice that are obese because of the lack of leptin	EPS is a potent gene expression regulator (in ob/ob mice) in obesity, insulin resistance and DM. Ferritin and adiponectin as important factors for future DM2.	33
		Expression level of Apo A-I, IV, C-III, E, retinol-binding protein 4 and transferrin were shown to be altered and their levels are normalized after EPS treatment.	
		Resistin is up-regulated while adiponectin is down-regulated in diabetes and obesity.	
2DE-DIGE MALDI-TOF	Adipose tissue	9 higher expressed proteins in the adipocytes from old compared to young obese patients:	92
		Prohibitin 1	
		Protein disulphide isomerase A3	
		Beta actin	
		Profilin	
		Aldo-ketoreductase 1 C2	
		Alpha crystallin B	
		Anexins A1, A5, A6	
		4 lower expressed proteins in the adipocytes from old compared to young obese patients:	
		Keratin type 2 cytoskeletal 1	
		Keratin type 2 cytoskeletal 10	
		Haemoglobins A, B	
		Signal transducer and activator of transcription 3 as the central molecule in the connectivity map and the apoptosis pathway	
iTRAQ LC-MS/MS	Heart tissue	29 proteins up-regulated from a total of 1.627, while 84 were down-regulated in the db/db mice compared with the control group	93
		Calnexin was found to be decreased whereas integrin-linked protein kinase was decreased in the phlorizin treated DM group compared with the DM group	
SDS-PAGE LC-MS/MS/MS LTQ-FT ELISA	Subcellular fractionation of the mouse preadipocyte cell line 3T3-L1 with and without insulin treatment into cytosol, membrane, mitochondria and nuclear fractions and nuclear fractions	3.287 identified proteins that form part of the adipocyte proteome	94,95
	Genetically modified animal models (bGH, GHA and GHR−/− mice and tissue-samples	Useful information to unravel the complexity of the adipocyte in obesity	
		Addressed that adiponectin is generally negatively associated with GH activity, regardless of age	
		Useful information about the associations of total and HMW adiponectin with insulin sensitivity and longevity	
		Circulating adiponectin levels correlated strongly with inguinal fat mass, implying the effects of GH on adiponectin are depot-specific	
Phosphoproteomics SILAC anti-pY immunoprecipitation	Brown adipocytes	From the 40 insulin effectors identified, 7 (SDR, PKC binding protein, LRP-6 and PISP/PDZK11, a potential calcium ATPases binding protein	96
2DE-gels stained by Sypro-Ruby	Platelet-free plasma from the patients	53 differentially spot-proteins from which 51% were shown to be down-regulated comparing Vit D deficiency	97
		The HMW form of adiponectin is down-regulated in obese paediatric patients with Vit D deficiency	
		Thrombospondin 1 (TSP1) is up-regulated while histone deacetylase 4 (HDAC4) is down-regulated	
SCX MS/MS	Peripheral blood mononuclear cells	TSP1 and HDAC4 recover their normal expression level due to physical exercises	98
2DE-gels LC-MS/MS MALDI-TOF MS	Liver sample	A diet rich in n-3PUFA decreases the expression of regucalcin, aldehyde dehydrogenase	99
		A diet rich in n-3 PUFA increases	
		the expression of a POLI protein-A-1, S-adenosylmethionine synthase, fructose 1,6 biphosphatase, ketohexokinase, malate dehydrogenase, GTP-specific succinyl CoAsynthase, Ornithine aminotransferase, protein disulfide isomerase A3	
2DE-gels MALDI-TOF MS and MS/MS	Human subcutaneous (SQ) and white adipose tissue (WAT)	The levels of several proteins in human SQ-WAT are not homogeneous between different WAT depots	100
		Twenty-one proteins showed differential intensities among the six defined anatomical locations, and 14 between the superficial and the deep layer (such as vimentin, heat-shock proteins, superoxide-dismutase, fatty acid-binding protein, alpha-enolase, ATP-synthase among others)	
2DE-DIGE MALDI-TOF MS and MS/MS	Visceral adipose tissue (VAT) from pre-obese diabetic patients	The presence of diabetes influences the VAT abundance of several proteins	83
		Diabetic patients showed increased VAT abundance of glutathione S-transferase Mu 2, peroxiredoxin-2, antithrombin-III, apolipoprotein A-IV, Ig κ chain C region, mitochondrial aldehyde dehydrogenase and actin, and decreased abundance of annexin-A1, retinaldehyde dehydrogenase-1 and vinculin, compared with their non-diabetic counterparts.	
Label-free quantitative proteomics	Salivary samples from patients with diabetes	This study demonstrates that differences exist between salivary proteomic profiles in patients with diabetes based on the A1C levels	84
2DE MALDI-TOF MS and MS/MS	Serum samples from obese children	This research study establishes the bases of the utility of proteomics to assess clinical improvements in obesity.	85
		*Down-regulated proteins in obese patients: transthyretin apolipoprotein-A1, apo-J/clusterin and vitamin D binding protein.*	
		*ApoA1 was further down-regulated under the presence of up-regulation insulin resistance, whereas weight reduction induced its up-regulation.*	
		Apolipoprotein-A1 and haptoglobin were validated *via* ELISA as true potential candidate biomarkers	

Representative assays detailing in each column –the goals, technologies, type of sample to be analysed and the resulting data– are placed schematically in this table. 2DE-electrophoresis is one the most common tools used in diabetes and obesity research studies when using proteomics. Nevertheless, currently, more scientific articles are appearing and showing the advantages when applying HPLC or nano-HPLC coupled directly to mass spectrometry (LC-MS) to avoid losing low expressed proteins or putative biomarkers. Biomarkers, adipocyte and insulin proteomes have been the most common goals followed by scientists to unravel diabetes and obesity pathologies. All of them –and many others– allowed us to advance and establish the right platforms and current technology-innovations will permit improve diagnoses and refine therapies *via* identifying new biomarkers by proteomics.

There are many steps that can be taken in protein handling and storage processes, which may help to minimize any damage and, in turn, maximize accuracy of results. Crucial steps in the process are to use anti-proteases and anti-phosphatases, and to freeze the protein sample quickly after harvesting in several vials. A very common premise is that ‘there are no routine sample preparation-protocols’ for a given proteomic experiment; it is always necessary to optimize them according to the clinical goals and according to the designed proteomic strategies to be used. In any event, optimized protocols for a given sample or samples from the same clinical assays can be used routinely. Moreover, several international laboratories [from Human Proteome Organization (HUPO), EUPA and SeProt] are standardizing specific protocols for storing clinical samples apart from those for analysis *via* proteomics and MS using reproducible protocols from different laboratories. This is important when we carry out analyses and the data are coming from different experiments from different laboratories 13–15.

Although the amount of protein obtained from patient samples can be very low, clinical samples allow highly valuable data to be achieved even with a limited amount of samples. Once the type of sample to be used has been selected, it is necessary to optimize the protocol for tissue disruption/cell-lysis or for the isolation of the proteins from body fluids using commercial reagents including protease and phosphatase inhibitors. When analysing serum/plasma, it may be necessary to use an albumin/IgG depletion kit to identify proteins whose concentration is low 15.

We have found that many proteins do not exist as a single form but as isoforms probably generated by post-translational modifications (PTM) of a given protein. Thus, for any proteomic protocol used, one must be knowledgeable about protein isoforms. In certain instances, it is also recommended that the purified complex mixture of proteins be dilapidated. Additionally, one often analyses a given tissue along with serum or urine. This type of complementary data may or may not be easily interpreted but many clinical research studies show that analysing both types of samples offers the possibility to explain linked biological processes. Additionally, the selection of a given proteomic platform must be made according to the goal of the experiment 16,17. For example, high-throughput–proteomic strategies may be useful for discovery of biomarkers specifically related to various stages of obesity and DM2, and they may help to improve current treatments and innovative therapies. On the other hand, once the target proteins are selected by the discovery phase of the research, analysis of those proteins in the complex mixture of biological samples can be conducted by selected reaction monitoring (SRM) or multiple reaction monitoring (MRM) assays. The sample-preparation of body fluids is a tedious but critical step in obtaining reliable results 16–19. For example, urine contains low levels of proteins compared to blood and a large amount of sample and/or a concentration procedure may be required for analyses. The timing of the sample collection may influence the results because the protein composition of body fluids may be different according to the different stages of the treatment for each patient. Indeed, we hypothesize that the serum from a patient suffering DM2 may contain different protein patterns at diagnoses (t0) during the treatment (t1) and also at the end of the therapy (t2). Nevertheless, there is no reference protein-map for DM2 or for obesity; thus, if we are able to ‘build’ the protein-pattern reference map of obesity and DM2 compared to healthy controls, we should improve our understanding of these pathologies.

Clinical application of proteomics, applied according to space (serum, urine, blood) and time (diagnoses, treatment and/or cured states), can be used to study the evolution of patients suffering obesity and DM2, to unravel the mechanisms involved in disease progression. In the end, we hope that the patients will benefit.

In the following sections, we discuss specific details useful for carrying out obesity and DM2 proteomic research.

## Special issues concerning tissue/blood sampling

As stated above, sample integrity and storage are key factors for obtaining efficient and reproducible data. Within the last decade, the quantity and quality of stored samples are high because of the creation of biobanks including sample collection procedures. This implies that the samples are kept intact, preserving their chemical and physical characteristics that may ultimately result in data indicative of their functions and/or roles within the cell. Thus, different types of samples (blood, urine, tissues) from the patients with different pathologies are available for study 18,19 or can be collected and properly stored in an individual laboratory’s biobank.

Another important step is related to the standardization of protocols for data acquisition from different biobanks so that different disease states and/or changes throughout time can be compared 20. If one of the main goals is to establish a long-term repository of biological/clinical samples and to make these samples available to multiple scientific studies, then specific protocols must be used. For example, if we have a particular interest in diabetes, fasting samples should be collected and stored. Additionally, a patient’s phenotypic data must be recorded including age, sex, weight, BMI, fasting glucose and other clinically important parameters. These data should be obtained at diagnoses and at different states throughout the disease. Protocols to store clinical samples should provide clear information on how and when the samples were collected, processed and organized to ensure their long-term integrity for the study of a given disease 21.

### Serum samples

One of the major difficulties in studying the serum proteome is that two major groups of serum proteins, albumin and immunoglobulins, comprise approximately 95% of the total, while the remaining 5% belongs to a variety of other protein types including cytokines, hormones, enzymes and cytoplasmic and nuclear proteins. Those highly abundant proteins may generate substantial background in any proteomic analysis and may mask the significant changes in proteins of interest. Albumin and IgG can be removed from serum using affinity chromatography to refine the identification of biomarkers (low expressed proteins) for a given pathology 22. However, since albumin interacts with many blood components, it is possible that by removing albumin, one may inadvertently remove important proteins that are important to a given pathology, in this case DM2.

The lipids contained in serum can have an important role in specific pathologies, such as hyperlipidemia, cardiovascular diseases, *etc*., as they can interact with serum proteins. In some cases, it is necessary to remove these lipids to identify the required proteins. Fractionating the serum *via* centrifugation allows one to then separate chylomicrons, very low density lipoproteins (such as VLDLPs) from the total protein sample 23–25.

Results of proteomic analyses comparing a diseased *versus* healthy state and the different stages of the disease can ultimately identify putative therapeutics and/or therapeutic targets as well as diagnostic biomarkers. It has been suggested that the concentration of specific proteins in serum can be useful for the early detection of insulin resistance and that this may even be possible before the appearance of the symptoms 23,24,26. Thus, identification of differentially expressed serum proteins could be potentially important for the prevention and treatment of DM2, even in obese children 26–28.

## Proteomic methodologies

The most significant breakthrough in proteomics has been the use of MS for the identification of proteins directly obtained from clinical samples or from samples in which the proteins were previously separated by two dimensional (2DE) gel electrophoresis or chromatography. Typical MS based identification of a protein utilizes tryptic digestion 29 prior to MS analysis to improve the accuracy or mass measurements. Mass spectrometry ionizes protein or peptide samples and measures their mass-to-charge ratios. Two ionization methods often employed are matrix-assisted laser desorption/ionization (MALDI) and electrospray ionization (ESI). The time –of –flight (TOF) analyzer is often used with MALDI. The TOF analyzer accelerates the ions in an electric field and measures the time they take to reach the detector. If the sample’s charge is the same, velocity is inversely related to their masses. The quadrupole mass filters are used with ESI. It consists of four parallel metal rods. By controlling electric field within the four rods by a radio frequency voltage, only ions with a certain mass-to-charge ratio can be selected and reach the detector. The quadrupole ion trap works similar to the quadrupole filter, but instead of passing a single ion species through, it traps ions and releases them sequentially.

Tandem MS is an array of MS that enables peptide sequencing. The first MS isolates a peptide to be analysed and the second MS fragments the peptide by collision-induced dissociation (CID) by a neutral molecule such as nitrogen. Finally a third MS measures mass-to-charge ratio of the resulting fragments 30. A series of three quadrupoles can be used in the same manner. Since CID fragments a peptide randomly at the peptide bonds, sequence information can be obtained by analysing the resulting mass spectrum.

Mass spectrometry can be coupled to chromatography. For selected proteomic analyses, liquid chromatography (LC) is often used to separate proteins or peptides, then masses of the separated peptides are determined by MS. For LC-MS analyses only microgram quantities of a sample are necessary for its characterization 31,32.

The 2DE gel-based technique is a very useful tool for protein separation 33, where during the first dimension proteins are separated according to their net charge by isoelectric focusing *via* immobilized pH gradient gel-strips. Subsequently, during the second dimension, proteins from the gel-strips are resolved according to their molecular weight using SDS-PAGE. 2DE can be very useful as post-translationally modified protein isoforms are typically revealed. These protein isoforms are not easily resolved by the other methods. A drawback of 2DE is related to gel-to-gel variations and the necessity of a large amount of samples (usually from 300 μg to 1 mg) for analysis. Moreover, the detection limit of proteins in 2DE is in the microgram range. In spite of this, interesting 2DE studies have resulted in important data pertaining to DM 34.

In addition, 2DE-gels have been coupled to differential in-gel electrophoresis (DIGE). In 2DE-DIGE, the protein samples are labelled with fluorescent dyes and then separated by 2D-PAGE. Different biological samples are labelled with different fluorescent dyes, mixed together and separated in the same gel. The gels are scanned by laser and the images of 2D-PAGE gels for multiple samples are obtained from the single gel. 2D-DIGE overcomes gel-to-gel variations and enables comparisons of multiple samples directly. Although 2DE-DIGE is a more refined strategy than 2DE, the detection limit remains at 0.5 μg.

In contrast to 2DE, LC-MS is a gel-free tool where digested proteins or tryptic peptides can be separated according to their physical and chemical properties (*i.e*. hydrophobicity, charges and pH). LC-MS has a higher sensitivity than 2DE-gels allowing the detection of proteins present at low levels and at the same time highly expressed proteins. Nevertheless, high abundant proteins such as some serum proteins (albumin) must be depleted to refine the resulting data 34.

Ion exchange chromatography is a popular methodology that allows the purification of proteins as well as other charged molecules. In strong cation exchange chromatography (SCX) positively charged molecules are attracted to a negatively charged solid support. Conversely, in strong anion exchange chromatography (SAX), negatively charged molecules are attracted to a positively charged solid support. Ion exchange chromatography consists of a methodology to separate molecules based on differences related to their accessible surface charges. This technique is extensively applied in the pre-fractionation and/or purification of target protein(s) from crude biological samples. It involves the reversible adsorption of charged molecules to immobilized ion groups on a matrix of an opposite charge, thus, interactions among molecules and active-sites on the membrane support, occur in a convective manner *via* pores. Once the sample is loaded and equilibrium is reached, the molecules reach the adsorption step because of appropriate charge and displace the counter ions. Finally, they bind reversibly to the matrix. Generally, the unbound materials will pass by the column with the void volume. In the third stage, *via* increasing the ionic strength of the eluting buffer, substances are removed from the column. Also, high protein recovery rates with intact biological activity are produced by the relatively mild binding and eluting conditions of this separation method. Furthermore, SCX and SAX can be easily coupled to MS 35,36.

## Analysis of protein phosphorylation

It is very important to analyse phosphorylated proteins in clinical research, as they may imply new targets for drug/therapy innovations. We will detail it in a simple manner *via* the most currently useful tools.

### Sequential elution of (IMAC) followed by TiO_2_

Immobilized metal affinity chromatography (IMAC) and titanium dioxide (TiO_2_) are metal-beads that can be packed to construct manual metal-affinity chromatography-tips to isolate primarily multi-phosphopeptides *via* IMAC and mono-phosphopeptides *via* TiO_2_ 35. Thus, this tool is useful for the isolation of mono and multi-phosphorylated protein/peptides from a biological complex-analyte in a single experiment. The basic principle relies on the fact that metals contained in IMAC and TiO_2_ carry positive charges and link the negatively charged-phosphopeptides. TiO_2_, IMAC and ZrO_2_ are all useful for phospho-enrichments, but Thingholm *et al*. 37 demonstrated a higher yield from SIMAC when purifying mono and multi-phosphopeptides in a single experiment; thus we recommend SIMAC as it works well for clinical samples. The most common techniques for enrichment for individual and/or global phosphorylation are IMAC and TiO_2_ 38, which are based on the high affinity of positively charged metal ions. However, conversion of carboxylate groups to esters effectively eliminates non-specific retention of non-phosphorylated peptides, although this constitutes a drawback because of the increased complexity in the subsequent MS analysis.

During the last 10 years, TiO_2_ has emerged as the most common of the metal oxide affinity chromatography based phosphopeptide enrichment methods. This technique offers increased capacity compared to IMAC resins to bind and elute mono-phosphorylated peptides. TiO_2_ exploits the same principle as IMAC, and is similarly prone to non-specific retention of acidic non-phosphorylated peptides. However, when loading peptides in 2,5-dihydroxybenzoic acid 39, glycolic and phthalic acids, non-specific binding to TiO_2_ is reduced, thereby improving phosphopeptide enrichment without a chemical modification of the sample. TiO_2_ is often considered to be interchangeable with IMAC. It works on similar levels of sample quantity (*e.g*. micrograms of protein) for the identification of phosphorylation-sites by MS analysis. Recently, SIMAC 39–41 appeared as a phosphopeptide enrichment tool that exploits the properties of IMAC coupled to TiO_2_, making it possible to carry out more refined studies.

Another phosphopeptide enrichment procedure prior to MS analysis is ZrO_2_ 42 and its principle is based on metal affinity chromatography like IMAC and TiO_2_. ZrO_2_ permits the isolation of single phosphorylated peptides in a more selective manner than TiO_2_. It has, in fact, been successfully used in the large-scale characterization of phosphoproteins 43–46. Furthermore, strategies that consist of fractionating and subsequently enriching phosphopeptides are based on strong cation/anion exchange (SCX and SAX) chromatography and HILIC interaction chromatography. Calcium phosphate precipitation is also a useful pre-fractionation step to simplify and enrich phosphopeptides from complex samples which can be coupled to IMAC 46.

### Isobaric tag for relative and absolute quantitation

This technique allows the relative and absolute quantification of 2 to 8 complex-samples at the same time 47. It uses a multiplexed isobaric chemical tagging-reagent. This tag permits multiplexing of 2 to 8 complex-protein samples and produces identical MS/MS sequencing ions for all 8 versions of the same derived tryptic peptide. Quantification and analyses are performed *via* comparing the peak-areas and peak-ratios in the MS/MS mode. The reporter-ions used are from 114 to 117 Da and from 113 to 119 and 121 Da. To carry out protein expression level studies, it can be a good option to apply isobaric tag for relative and absolute quantitation (iTRAQ), even though stable isotope labelling by amino acids in cell culture (SILAC, when using cell lines) and/or MRM or SRM can also be successfully employed (see below) 48,49.

SILAC is another approach for *in vivo* incorporation of a label into proteins for subsequent MS-quantitative analysis. The identification step is currently carried out in MS/MS mode. Its principle is based on metabolic incorporation of a given ‘light’ or ‘heavy’ form of the amino acid into the proteins. Amino acids are incorporated *via* substituted stable isotopic forms such as deuterium, 13C and 15N. Basically, during SILAC experiments, two cell-populations are grown in a culture media that is identical except that one contains the ‘light’ label and the second one the ‘heavy’ label. These labels allow the comparison of two physiological conditions (*e.g*. healthy *versus* ill). Two conditions are distinguished *via* the labelled analogue amino acid that was added to the culture media. The newly synthesized proteins within cells have the same physical and chemical properties except for the mass differences as a result of the isotope 50.

### Selected reaction monitoring or multiple reaction monitoring

**Figure 1:**
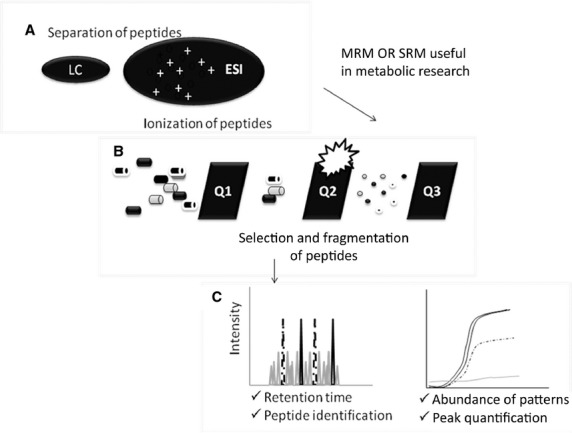
Scheme of SRM/MRM useful for metabolic research. SRM or MRM for quantitative assays consists of: (A) following, for example, ionization ESI type, (B) a peptide precursor is first isolated to obtain a substantial ion population of mostly the intended species. This population is then fragmented to yield product ions (C) whose signal abundances are indicative of the abundance of the peptide in the sample. SRM can be carried out on a triple quadrupole, where mass-resolving Q_1_ isolates the precursor, Q_2_ acts as a collision cell and mass-resolving Q_3_ is cycled through the product ions which are detected upon exiting the last quadrupole. A precursor/product pair is often referred to as a transition.

This is a quantitative proteomics MS/MS-based tool. When using ESI, first, a peptide precursor is selected and isolated to obtain an ion population that represents ‘the precursor’. Next, the ion population is fragmented to produce product ions or ‘daughter ions’. The signal-intensity of the fragmented ions represents the abundance of peptides and/or proteins for a given sample. Indeed, this method allows for absolute quantitative data. Selected reaction monitoring or MRM is carried out in specific mass spectrometers called ‘tripe quadrupole’ and ‘ion traps’. The sensitivity and specificity of such strategy is extremely high. In SRM assays, two mass analyzers are used as static mass-filters to monitor a particular fragment-ion from a selected precursor-ion. The resulting selectivity from the two filtering stages coupled to the high-duty cycle data in quantitative assays allows for extremely high sensitivity 51–60 (Fig.1 shows in a simple scheme of SRM/MRM useful for metabolic research.

### Label-free quantification

**Figure 2:**
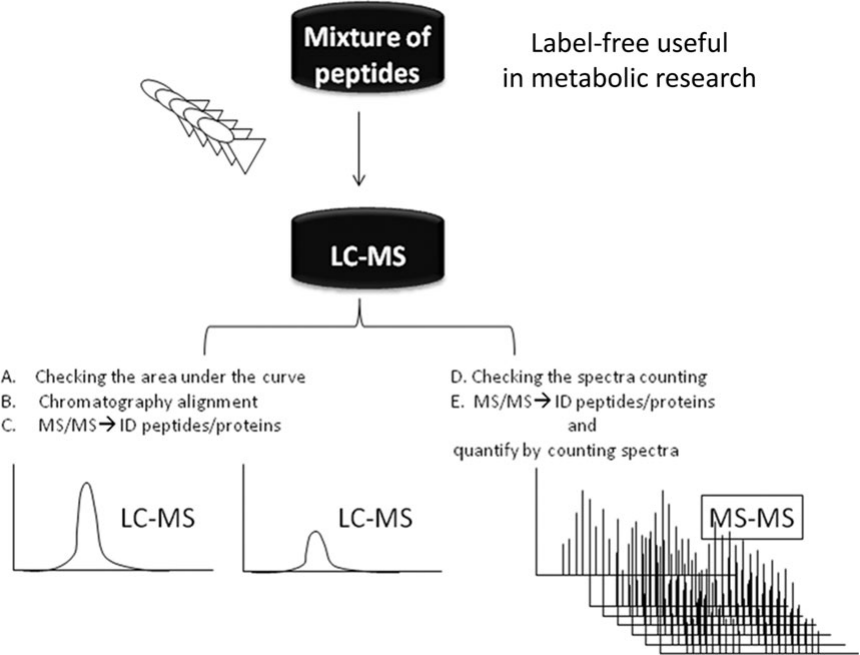
We suggest to perform this scheme of Label-free quantification useful for metabolic research. (A) Peptide signals are detected at the MS1 level and are distinguished from chemical noise/background by their characteristic isotopic pattern. (B) These patterns are then followed *via* the retention time dimension and are used to rebuild a chromatographic elution-profile of the mono-isotopic peptide mass. (C–E) The total ion current of the peptide signal is then integrated and used as a quantitative measurement of the original peptide concentration. For each detected peptide, all isotopic peaks are first found and the charge state is then assigned. Label-free can be carried out *via* Fourier Transform Ion Cyclotron Resonance (FTICR) or Orbitrap.

This is a MS-based method that allows the determination of the relative amount of protein from 2 or more complex samples 61. Label-free quantification does not use a stable isotope containing compound to chemically bind to the proteins. In a label free quantitative proteomic analysis, protein mixtures are analysed directly and samples are compared to each other after independent analyses. As a result, there is no mixing of samples, so that higher proteome coverage can be achieved and there is no limit to the number of experiments that can be compared 62. Moreover, label-free approaches may be divided into two main groups by the way that the abundance of a peptide is measured. The first group comprises methods that are based on the ion count and compare either maximum abundance or volume of ion count for peptide peaks at specific retention times between different samples. Since ionized peptides elute from a reversed-phase column into the mass spectrometer, their ion intensities can be measured within the given detection limits of the experimental setup 43–45,63–68 (Fig.2 shows in a simple manner the scheme of Label-free useful for metabolic research).

### Electrospray ionization and tandem mass spectrometry MS-n (Nano-ESI-MSn)

Tandem MS 69, ESI coupled with CID and MS/MS potentially represents one of the most sensitive, discriminating and direct methods for the qualitative and quantitative high-throughput analysis of sub-picomole amounts of protein. Nano-ESI-MS/MS allows identification of the previously labelled and isolated proteins/peptides (and phospho-proteins/peptides) coming from complex samples (*i.e*. blood and/or CSF) and subsequently, data analyses of the quantified (from iTRAQ label) and modified residues (from isolation SIMAC) must be conducted *via*, for example, Matrix-Science Mascot searching (http://www.matrixscience.com/cgi/search_form.pl?FORMVER=2&SEARCH=MIS).

Through the previously mentioned tools, we can achieve: (*i*) identification (ID) of protein candidate biomarkers, (*ii*) analyse how the intracellular signalling -networks are activated/deactivated *via* phosphorylation during disease progression and (*iii*) determine which proteins and phosphoproteins or other PTMs are up and/or down-regulated in several clinical states (healthy or ill). The resulting data is specific for a given time and/or disease state (*i.e*. after diagnosis, during treatment) of a specific patient/s and according to each different type of sample (blood, sera, urine) as the proteome is dynamic (space and time). Finally, the resulting identified protein-biomarkers can be validated by ELISA and/or western blotting and *via* SRM/MRM assuming that an antibody is available for a given protein 70–72. To summarize, to obtain reliable and reproducible biomarker data, it is always necessary to standardize protocols for the collection, handling, storage and processing of samples for subsequent proteomic analyses using any of the procedures described above. These tools allow the analysis of hundreds of proteins at a given time in a very small sample size with high sensitivity. The main difference among these current techniques is related to the sensitivity, the detection-level of the selected method 73–80.

## Identification of proteins

Mascot Server (http://www.matrixscience.com/) is commonly used for identification, characterization and quantification of the resulting proteins after MS results have been collected. It allows free searches and contains protein sequence databases online from all organisms analysed to date. Through Mascot it is also possible to validate the identified protein-biomarkers from the clinical proteomic research study *via* manual inspection of all the spectra. Manual validation of spectra is required by the best proteomic journals to ensure the high quality of data. New and efficient bioinformatic software is routinely appearing on the market to carry out statistics and validation, especially for high-throughput analysis; thus, the validation-step can be developed more efficiently. Interesting and useful bioinformatic tools for proteomics research are well-established in other reviews 80,81.

**Figure 3:**
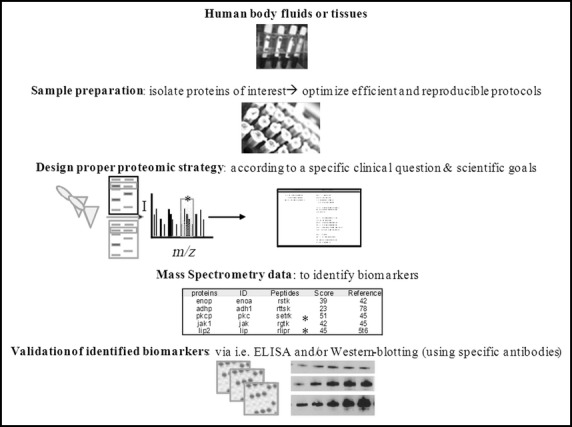
General scheme of current proteomic-flow trough, for clinical metabolic research. Human body fluids (*i.e*. sera, urine and blood) have to be properly stored and prepared with optimised protocols. Subsequently, the proteins should be purified and/or isolated to get digested peptides (*i.e*. using trypsin). The adequate proteomic-MS based strategy is applied, and once we get the data (potential biomarkers), validation assays (*i.e*. ELISA and/or western blotting) can be carried out choosing specific antibodies to identify real protein-biomarkers. Currently, clinical proteomics research involves high-performance chromatography coupled to mass spectrometry avoiding 2DE-gels to identify high and low abundant proteins in a given clinical sample.

Thus, it is obvious that the methodology available to perform proteomics and phosphoproteomic studies has advanced dramatically in recent years, to improve our understanding of intracellular signalling networks. These methodological advances are now beginning to be applied to studies of DM2 and the identification of new biomarkers. Figure3 shows, in a simple manner, the basic work-flow useful for metabolic research (Fig.3). Below we briefly review some of the most relevant studies in diabetes using proteomic approaches.

## Current and relevant studies of DM and obesity using proteomic approaches

Yang *et al*. 82, carried out 2DE of peritoneal dialyzed samples. They were able to visualize more than 300 protein-spots, from which 13 protein spots were selected to be excised and the corresponding proteins were digested and analysed by reverse-phase nano-ultra performance LC-ESI-MS/MS. The resulting data confirmed 10 spots/proteins with significant differential expression between the DM and chronic glomerulonephritis peritoneal dialyses samples. These authors state that the differentially expressed proteins identified in their study may not be new biomarkers. However, they also indicate possible targets for drug treatment and protein profiles that could be predictors of peritonitis, indicating the necessity for validation studies in the future. A possible way to validate the resulting identified protein-biomarkers from obesity and DM2 samples during clinical proteomic research studies is *via*: (*i*) ELISA and/or western blotting (using specific antibodies (Abs) for the identified potential biomarkers-) and (*ii*) *via* SRM/MRM 4. Selected reaction monitoring or MRM is a method of MS/MS that allows one to monitor target peptides within a complex mixture of tryptic peptides. For example, proteins A1 and B2 are discovered as biomarkers for DM2 at diagnosis in obese patients, while proteins C3 and D4 are biomarkers for DM2 during treatment with complications and proteins E5, E6 and E7 are indicators of good prognosis. These candidate biomarkers can be routinely monitored in each patient to get more information about how to improve the treatment, regime and their prognosis.

In 2009 Kim *et al*. 83 carried out proteomic analysis in ob/ob mice, which lack leptin, before and after hypoglycaemic polysaccharide treatments. Their goal was to identify biomarkers of DM2 prognosis using 2DE-gel electrophoresis (2-DE). Specifically, these authors studied the influence of hypoglycaemic extracellular polysaccharides (EPS) coming from the macrofungus *Tremella fuciformis* on the differential levels of plasma proteins in *ob/ob* mice *via* 2-DE. They were able to visualize 900 spots of which 92 were differentially regulated in ob/ob mice. From these 92 spots, 40 were identified to be relevant diabetes-associated proteins. Furthermore, they were able to corroborate that high serum level of ferritin, which acts as an antioxidant *via* binding the iron-excess, is a risk factor for future-DM2, and that adiponectin plays an important role in the metabolic syndrome and inflammation, as low serum levels of adiponectin are a risk element for DM2. They also demonstrated that Apo A-I, IV, C-III, E, retinol-binding protein 4 and transferrin proteins were significantly altered in ob/ob mice, and their levels were normalized after EPS treatment. Through western blot, they observed that while resistin is up-regulated, adiponectin is down-regulated in diabetes and obesity and this was normalized with EPS. Moreover, to complement the resulting proteomics data, Kim *et al*. 83 investigated the differential gene expression patterns in different tissues, such as liver, adipose tissue and muscle of ob/ob mice in response to EPS treatment, by use of PCR arrays. The resulting data demonstrated that the expression level of many genes related to the onset, development and progression of diabetes was significantly down-regulated by EPS therefore suggesting that EPS might act as a potent regulator of gene expression for a wide variety of genes in ob/ob mice, particularly in obesity, insulin resistance and complications from diabetes mellitus 83. Thus, Kim *et al*. 83 successfully coupled different OMIC tools to improve and corroborate the resulting data.

It is well-known that obesity and ageing affect adipocyte metabolism and the distribution of fat in subcutaneous and visceral depots. Moreover, weight gain and ageing can lead to similar clinical outcomes as, for example, insulin resistance, cardiovascular disease and atherosclerosis. Alfadda *et al*. 84 studied the expression level of proteins in obese patients in relation to their subcutaneous adipose tissues and age. Through 2DE-DIGE coupled to MALDI-TOF these authors were able to identify 9 highly expressed proteins and 4 lower expressed proteins in adipocytes from old compared to young obese patients. Some of the more highly expressed proteins include: (A1) prohibitin 1, (A2) protein disulphide isomerase A3, (A3) beta actin, (A4) profilin, (A5) aldo-ketoreductase 1 C2, (A6) alpha crystallin B and (A7) the annexins A1, A5 and A6. The 4 less abundant proteins are: (B1) keratin type 2 cytoskeletal 1, (B2) keratin type 2 cytoskeletal 10 and (B3) haemoglobins A and B. These are involved in regulation of apoptosis, cellular senescence and inflammatory responses, all of which are common pathologic events in obesity and ageing. In addition, signal transducer and activator of transcription (STAT) 3 was identified as the central molecule in the connectivity map and the apoptotic pathway as the pathway with the highest Mascot score: (Mascot is a search engine which uses MS data to identify proteins from primary sequence databases, as the resulting digest peptide mixture is analysed by the sequence from MS as described above) (http://www.matrixscience.com/search_form_select.html). Differences in the abundances of prohibitin 1, protein disulphide isomerise A3, beta actin, profilin and STAT3 proteins were validated *via* immunoblotting. The data resulting from the studies by Alfadda *et al*. demonstrated that the proteins identified to be differentially expressed in aged obese *versus* young obese patients suggest an increase in resistance to apoptosis as a defence mechanism against a state of chronic low-grade inflammation. They also state that their data provide clues for unravelling the biochemical mechanisms underpinning obesity and obesity-related ageing 84.

Recently many researchers have begun to use MS-based quantitative tools (*i.e*. iTRAQ) instead of 2DE or 2DE-DIGE. The main advantage is that MS-based quantification allows measurements *via* adding isotopes labels; thus more proteins, including low expressed proteins, can be quantified. Mass spectrometry-quantitative studies using iTRAQ in DM2 represent a good possibility for meeting the challenge of improving the data collected to date, as it belongs to a very refined proteomic strategy.

A relevant example is the study of Cai *et al*. 85 who analysed cardiomyopathy and DM2 with the iTRAQ proteomic approach. Diabetic cardiomyopathy accompanying T2DM is a complicated disorder caused by a multifactorial pathology including altered cardiac energy metabolism and increased oxidative stress. Obesity is associated with high levels of circulating fatty acids, which can result in increased fatty acid uptake and TG accumulation in the myocardium. Furthermore, increased oxygen damage and generation of reactive oxygen species (ROS) augment cardiac damage. Thus, normalization of cardiac energy metabolism and reduction in oxidative stress may be important factors in the treatment of diabetic cardiomyopathy. Phlorizin has been reported to have an anti-diabetic effect because of its antioxidant properties. Although phlorizin is an antioxidant used in treating DM, its cardio-protective effects on diabetic cardiomyopathy are not well established. Through iTRAQ high throughput proteomics Cai *et al*. studied the function of phlorizin in preventing diabetic cardiomyopathy in *db/db* mice, which are obese and diabetic because of a defect in the leptin receptor. They coupled LC-MS/MS to iTRAQ to identify and characterize the protein profiles of phlorizin-treated and untreated *db/db* mice. Heart tissue from treated and untreated mice was prepared for iTRAQ analysis to obtain the protein-profile pattern of phlorizin’s effect on myocardial proteins. A total of 1627 proteins were identified. Of the 113 differentially expressed proteins, 29 were elevated in the db/db group compared with the control group, but were decreased by phlorizin treatment. An additional 84 proteins were decreased in the *db/db* mice compared with the control group. The authors selected 2 proteins (calnexin and integrin-linked protein kinase) for western blotting analysis to validate the iTRAQ data. Calnexin was found to be decreased, whereas integrin-linked protein kinase was increased in the phlorizin treated DM group compared with the DM group. Quantification of the band intensity showed that the results were consistent with the iTRAQ data.

Cai *et al*. 85 were able to identify thousands of proteins of which 12–15% were differentially expressed. These proteins are involved in cardiac lipid metabolism, mitochondrial function and cardiomyopathy. The resulting data suggests that phlorizin may prevent the development of diabetic cardiomyopathy by regulating the expression of key proteins in these processes. They also showed that phlorizin significantly decreases body weight-gain and circulating levels of glucose, triglycerides, total cholesterol and glycosylated-end products. Their findings suggest that normal myocardial structure was better preserved after phlorizin treatment and that this drug can be a novel therapeutic protocol for the treatment of diabetic cardiomyopathy, with the proteomic data helping to identify the possible mechanism 85.

Adipocytes play a central role in energy metabolism and in the obesity epidemic. Therefore, determining the protein composition of adipocytes should help to unravel important biological questions. In such an endeavour, Mann *et al*. 86 studied the adipocyte-proteome by proteomics plus mass spectrometry and bioinformatic tools. They first carried out subcellular fractionation of the mouse preadipocyte cell line 3T3-L1 with and without insulin treatment, into cytosol, membrane, mitochondria and nuclear fractions. They used SDS-PAGE gels from which the protein-bands were digested and the resulting peptides were identified *via* LC-MS/MS/MS (LTQ-FT). They were able to identify 3287 proteins that form part of the adipocyte proteome. Moreover, each fraction was analysed by western blotting and using specific antibodies for each fraction. In addition, they validated all resulting data by bioinformatics, obtaining one of the largest high confidence proteomes reported. The adipocyte-proteome is available in the Max-Planck Unified Proteome database 86. This article contains very useful information to unravel the complexity of the adipocyte in obesity and other pathologies 86.

Another example from the group of Mann *et al*. 87 is related to unravelling the insulin induced intracellular signalling pathway *via* phosphoproteomics. It is well-known that the insulin signalling pathway is very important in metabolic diseases and cellular processes of ageing. The insulin receptor and its substrates are fundamental in the insulin signalling-network, with insulin binding to its receptor to trigger tyrosine phosphorylation cascades that subsequently activate other connected cascade networks. Understanding the activation of connected signalling networks is the key to unravelling diverse biological processes related to DM and obesity. To begin to understand the network of the tyrosine phosphorylation cascade, Mann *et al*. identified the tyrosine-phosphoproteome of the insulin signalling pathway by applying MS coupled to phosphotyrosine immunoprecipitation and SILAC in differentiated brown adipocytes. They also quantified the temporal dynamics of tyrosine phosphorylation events upon insulin stimulation in differentiated brown adipocytes. The authors applied high resolution quantitative MS-based proteomic tools (SILAC and anti-pY immunoprecipitation) to identify and quantify 40 protein effectors from the insulin pathway in differentiated brown adipocytes. To know the temporal dynamics of phosphorylation of proteins on tyrosine, the authors applied a triple label of SILAC with three differentially labelled cell populations being stimulated for different times. Data were corroborated by western blotting. From the 40 insulin effectors identified, 7 (SDR, PKC binding protein, LRP-6 and PISP/PDZK11, a potential calcium ATPase binding protein) were described for the first time to be involved in insulin signalling. In addition, Mann *et al*. indicate that this approach is capable of detecting the specific pY containing proteins/peptides-and quantify the level of their phosphorylation according to different stress and stimuli conditions over insulin network 87.

Walker *et al*. 88 have recently suggested that in obesity the ‘networks’ of metabolic signalling pathways are related to vitamin D status and that vitamin D regulation of adiponectin involves post-translational-events. They coupled 2DE-gels plus MS to identify relevant molecules in obese children dichotomized according to 25OH vitamin D (25OHD) levels. Platelet–free plasma from 42 obese children (M/F = 18/24) classified according to their 25OHD3 levels (<15 ng/ml = deficient and >30 ng/ml = non-deficient) was analysed. Image analysis (ChemiDoc Imager and PD Quest –software to analyse the resulting proteomic data-) was able to identify the protein-spots from 2DE-Sypro-gels that were differentially expressed according to each individual spot ‘volume’ *via* density/area integrating Sypro-Ruby staining to the Gaussian model. Their data showed 53 differentially expressed protein-spots, from which 51% were down-regulated (comparing VD deficiency and no deficiency of VD). One interesting identified biomarker is the HMW form of adiponectin, which was observed to be down-regulated in obese paediatric patients with vitamin D deficiency. Additionally, the identified biomarkers are able to be modulated *in vivo* with vitamin D supplementation. This proteomic approach represents a very interesting strategy to differentiate phenotypes of diseases and also to study specific therapy targets.

An important contributing factor for obesity and its associated comorbidites is a sedentary lifestyle, in addition to excessive food intake. Abu-Farha *et al*. 89 recently published an interesting study applying high-through-put proteomic analyses of peripheral blood mononuclear cells (PBMCs). The PBMCs samples were purified from lean and obese human males to identify and quantify differentially expressed proteins between both groups. In this study the mechanisms underlying obesity progression and the possibility of managing this progression with physical exercises were analysed. Using SCX plus MS/MS, 47 proteins differentially expressed between lean and obese patients were identified 89. Thrombospondin 1 (TSP1) was up-regulated while histone deacetylase 4 (HDAC4) was down-regulated and after 3 months of physical exercises, TSP1 and HDAC4 returned to control levels.

Omega-3 polyunsaturated fatty acids (n-3 PUFA) are reported to decrease the symptoms of diabetes, obesity and insulin resistance related to the metabolic disorders. The proteins and pathways involved in the regulation of n-3 PUFA are unknown, especially those which produce beneficial health effects. Ahmed *et al*. 90 studied the effect of diets with high or low levels of n-3 PUFA on hepatic proteomic profile in mice. They applied 2DE-gels coupled to LC-MS/MS analysis plus specific software (ImageScanner III; Progenesis Samespots, version 3.1). It is important to note that 800 μg of total protein needed to be loaded into 2DE-gels (it is now possible to use other proteomic tools, mentioned above, to avoid the necessity of using such a large amount of sample). The resulting protein-spots visualized *via* 2DE were digested with trypsin and analysed by MALDI-TOF (MS). They also carried out analysis by tandem mass spectrometry *via* LC-MS/MS to identify protein-spots from the 2DE-gels. MALDI-TOF MS is useful to study specific or a reduced number of protein-spots, while when using LC-MS/MS, a greater number of protein-spots can be more rapidly identified. They found that a diet rich in n-3 PUFA decreases the expression of regucalcin, adenosine kinase and aldehyde dehydrogenase. In addition, diets rich in n-3 PUFA increase the expression of apolipoprotein A-I, S-adenosylmethionine synthase, fructose-1, 6-bisphosphatase, ketohexokinase, malate dehydrogenase, GTP-specific succinyl CoA synthase, ornithine aminotransferase and protein disulfide isomerase-A3. Thus, these authors showed that n-3 PUFA produces modifications in many proteins related to regulation of lipids, carbohydrates, the citric acid cycle and protein metabolism and promoting the hypothesis that there is a network of metabolic pathways affected by n-3 PUFA 90 (Table1 shows examples of diabetes and obesity studies using proteomic tools: the type of sample and the clinical and proteomic goals are detailed, in addition the identified proteins/biomarkers, including the proteomic approach, are mentioned 91–102). Martos-Moreno *et al*. 103 evaluated the ability of serum proteomic analysis to detect the metabolic alterations compared to standard clinical assays, and also to identify potential new candidate biomarkers from metabolic impairment in very young obese children. They discovered that isoforms of apolipoprotein-A1, apo-J/clusterin, vitamin D binding protein and transthyretin were down-regulated *via* 2DE coupled to MALDI-TOF (MS, MS/MS) in young obese patients with some changes in these proteins being enhanced by insulin resistant and partially reversed after weight loss.

Interestingly, they observed that low molecular weight isoforms of haptoglobin were increased in obese patients, enhanced in insulin resistant obese children and again decreased after weight loss, being positively correlated with serum interleukin-6 and NAMPT/visfatin levels. The significance for a potential candidate biomarker was confirmed statistically for low molecular-weight isoform haptoglobin (obese *versus* control and insulin-resistant *versus* non-insulin-resistant) and Apo A1 (IR *versus* non-IR). Indeed, apolipoprotein-A1 and haptoglobin were validated *via* ELISA to confirm the clinical significance of those potential biomarkers related to metabolic complications in young obese children.

This research study establishes the base or substructure of proteomic utilities for obesity clinical improvements, to identify, in the near future, true candidate biomarkers may help to improve therapies at the early onset of obesity and insulin resistance in childhood 104.

List *et al*. 105 carried out proteomic analysis on the skin of C57BL/6J mice with type 2 diabetes using non-diabetic mice as controls. To induce obesity and diabetes, authors applied high fat diet to mice during 16 weeks. They applied 2DE and PDQuest software for the analysis getting around 1000 distinct protein spots. From those 1000 spots, 6 were shown to be significantly decreased while 22 were over-expressed in the diabetic state compared to controls. These analyses were carried in a MALDI-TOF in MS and MS/MS mode. List *et al*., observed that around 60% of the proteins that were up and/or down-regulated, belong to energy metabolism. The sample analysed in this study was diabetic skin, and it provides the identification of proteins from mouse-skin samples related to changes in obesity and subsequent diabetes. In addition, the authors remark on the relevance of skin biopsies coupled to proteomic assays as a very useful tool non-invasive for diagnoses of hyperinsulinemia and diabetes 105.

Kopchick *et al*. 104 remarked in at the beginning of 2009 on the use of proteomics in general for blood, urine and tissue analyses for the discovery of new growth hormone (GH) induced serum biomarkers. They discovered that isoforms of tranthyretin, clusterin, ApoE and ApoA1 are differentially expressed *via* MS and MS/MS coupled to 2DE-gels, thus these isoforms may be potential biomarkers for initiating studies using recombinant human growth hormone (rhGH). In addition, they suggest that with more biomarkers, it is possible to develop a robust, sensitive and specific test system for rhGH using a combination of multiple markers, thus achieving new targets and therapy improvements.

The detection of rhGH is difficult as it has a short half-life, thus, Kopchick *et al*. 106 indicate that novel and robust biomarkers of to detect rhGH abuse are needed. Through 2DE and MS, specific isoforms of alpha-1 antitrypsin and transthyretin were observed to be increased; while inter-alpha-trypsin inhibitor heavy chain H4, apolipoprotein A-1 and haemoglobin beta chain were observed to be decreased. The resulting data shows that high dose rhGH administration significantly reduced total serum protein concentrations and up- or down-regulated specific isoforms of five serum proteins. These protein isoforms may serve as potential biomarkers of rhGH treatment including when rhGH is misused and abused. For this study, serum samples- derived from the patients treated with rhGH in a randomized, double-blind, placebo-controlled-design were analysed. In addition, the biological meaning resulting from MS data was validated by western-blotting assays. Table1 summarizes useful metabolic research examples carried out *via* different proteomics strategies (Table1).

It is obvious that the current international human proteome sequencing programme (http://www.thehpp.org/human proteome project HUPO) will have an important impact on the diagnoses of diseases and the innovation of therapies 107–109. It has been predicted that the complete human proteome will be fully sequenced in less than 2–3 years; thus several chronic diseases, such as diabetes, obesity and/or ageing, will benefit from proteomics research 110. Therefore, reference map of the human proteome useful for many pathologies will be available, thus facilitating the use of proper controls for each clinical study 110, as it is very important to choose proper patients and good controls for independent verification, especially when looking for real protein biomarkers for improvements in diagnoses and therapies 111.

## Concluding remarks

The molecular mechanisms underlying obesity and its progression to DM2 are not completely known. Proteomics and other OMICs tools are helping to advance our understanding of the origin, onset, development, prevention and treatment of complex diseases including obesity and DM2. Moreover, as proteomics-MS-based technologies are becoming more sensitive and specific, employment of these tools is an important opportunity to augment our knowledge of DM2 and obesity and identify new targets for diagnosis and treatment.

Liquid chromatography-mass spectrometry results in increased accuracy compared to 2DE-gels, especially because of the fact that LC-MS allows identification of low expressed proteins (good protein candidate biomarkers). Thus, although important information has been achieved *via* 2DE (especially concerning isoforms studies), new concepts can be reached when analysing clinical samples *via* proteomic approaches that directly apply LC-MS avoiding 2DE.

The new possibilities of proteomic strategies, which include SIMAC, iTRAQ, label-free and nano-ESI-LC-MSn, could result in the identification of new phosphorylated biomarkers in the intracellular signalling networks involved in DM and obesity. It should be emphasized once more that correct clinical sample preparation is an essential pre-requisite and this should be standardized in biobanks.

**Figure 4:**
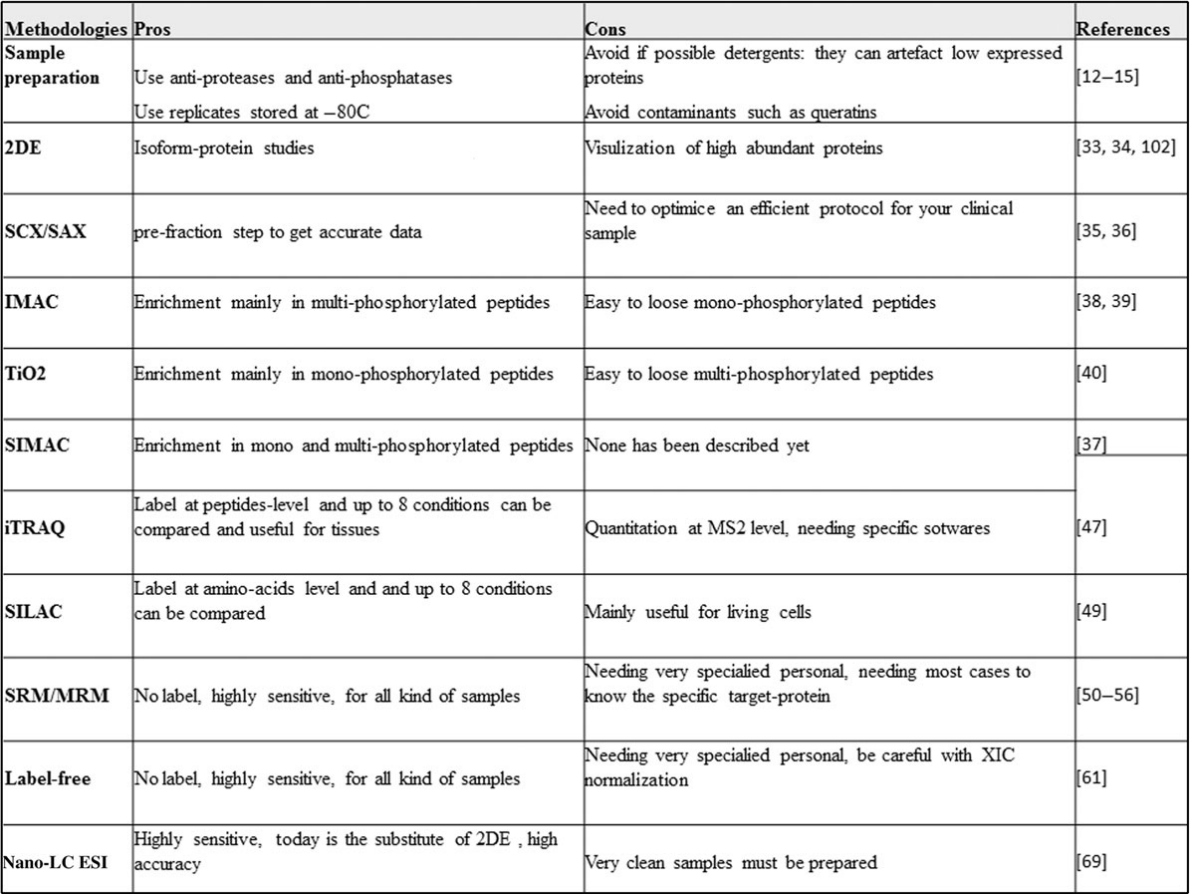
Pros and cons of several proteomic tools useful in metabolic research. In this figure, a summary of pros and cons (tips) of several proteomic methodologies, has been detailed, from sample preparation to get MS data. We aim to place useful tools for proteomic metabolic research.

To finalize, the advances in proteomic approaches and the complete sequence of the human proteome, will allow us to unravel changes in the proteomic profile of clinical samples from obese patients with and without diabetes, as well as DM2 with and without obesity (Fig.4 summarizes the pros and cons (tips) of several proteomic methodologies useful for metabolic research). Thus, it will benefit the patients and help to advance specific therapies. As a delegate of HUPO (http://www.hupo.org/; *Fostering international proteomic initiatives to better understand human disease*), for human proteome on children assays and studies at Hospital Universitario Niño Jesús (Madrid, Spain), we are seeking to support the human proteome in this context. We envision this will further benefit the understanding of the pathology of the diseases and ultimately improve the diagnoses and personalized treatments in the near future; thus, patients will undoubtedly be benefited.

## References

[b1] Martos-Moreno GÁ, Barrios V, Chowen JA (2013). Adipokines in childhood obesity. Vitam Horm.

[b2] Soriano-Guillén L, Barrios V, Martos G (2004). Effect of oral glucose administration on ghrelin levels in obese children. Eur J Endocrinol.

[b3] Nagaraj NS, Singh OV, Merchant NB (2010). Proteomics: a strategy to understand the novel targets in protein misfolding and cancer therapy. Expert Rev Proteomics.

[b4] López E, Madero L, López-Pascual J (2012). Clinical proteomics and OMICS clues useful in translational medicine research. Proteome Sci.

[b5] Anderson NL, Anderson NG (1998). Proteome and proteomics: new technologies, new concepts, and new words. Electrophoresis.

[b6] Blackstock WP, Weir MP (1999). Proteomics: quantitative and physical mapping of cellular proteins. Trends Biotechnol.

[b7] James P (1997). Protein identification in the post-genome era: the rapid rise of proteomics. Q Rev Biophys.

[b8] Wilkins MR, Pasquali C, Appel RD (1996). From proteins to proteomes: large scale protein identification by two-dimensional electrophoresis and amino acid analysis. Biotechnology.

[b9] DeLisi C (2008). Meetings that changed the world: Santa Fe 1986: human genome baby-steps. Nature.

[b10] DeLisi C (1988). The Human Genome Project. Am Sci.

[b11] Kussmann M, Morine MJ, Hager J (2013). Perspective: a systems approach to diabetes research. Front Genet.

[b12] López E, López I, Sequí J (2011). Discovering and validating unknown phospho-sites from p38 and HuR protein kinases in vitro by Phosphoproteomic and Bioinformatic tools. J Clin Bioinforma.

[b13] Silva JL, Vieira TC, Gomes MP (2010). Ligand binding and in protein misfolding: insights from studies of prion and p53 tumor supresor proteins. Acc Chem Res.

[b14] Murri M, Insenser M, Luque M (2014). Proteomic analysis of adipose tissue: informing diabetes research. Expert Rev Proteomics.

[b15] Takahashi E, Okumura A, Unoki-Kubota H (2013). Differential proteome analysis of serum proteins associated with the development of type 2 diabetes mellitus in the KK-A(y) mouse model using the iTRAQ technique. J Proteomics.

[b16] Chen X, Andersson R, Cho WC (2012). The international effort: building the bridge for Translational Medicine: Report of the 1st International Conference of Translational Medicine (ICTM). Clin Transl Med.

[b17] López E, Muñoz SR, Pascual JL (2012). Relevant phosphoproteomic and mass spectrometry: approaches useful in clinical research. Clin Transl Med.

[b18] Prakash A, Tomazela DM, Frewen B (2009). Expediting the development of targeted SRM Assays: using data from shotgun proteomics to automate method development. J Proteome Res.

[b19] Gemoll T, Löwe O, Borén M (2013). The impact of pre-analytical conditions on the serum proteome: heat-stabilization versus nitrogen storage. Arch Physiol Biochem.

[b20] Malm J, Fehniger TE, Danmyr P (2013). Developments in biobanking workflow standardization providing sample integrity and stability. J Proteomics.

[b21] Elliott P, Peakman TC (2008). The UK Biobank sample handling and storage protocol for the collection, processing and archiving of human blood and urine. Int J Epidemiol.

[b22] Dhokia B, Pectasides D, Epenetos AA (1986). Serum levels of c-myc and c-ras oncogene products in normal subjects and in patients with neoplastic and non neoplastic conditions. Int J Biol Markers.

[b23] Bhonsle HS, Korwar AM, Chougale AD (2013). Proteomic study reveals downregulation of apolipoprotein A1 in plasma of poorly controlled diabetes: a pilot study. Mol Med Rep.

[b24] von Toerne C, Kahle M, Schäfer A (2013). Apoe, Mbl2, and Psp plasma protein levels correlate with diabetic phenotype in NZO mice–an optimized rapid workflow for SRM-based quantification. J Proteome Res.

[b25] Chowen JA, Argente J, Horvath TL (2013). Uncovering novel roles of nonneuronal cells in body weight homeostasis and obesity. Endocrinology.

[b26] Okada S, List EO, Sankaran S (2010). Plasma protein biomarkers correlated with the development of diet-induced type 2 diabetes in mice. Clin Proteomics.

[b27] Granado M, García-Cáceres C, Tuda M (2011). Insulin and growth hormone-releasing peptide-6 (GHRP-6) have differential beneficial effects on cell turnover in the pituitary, hypothalamus and cerebellum of streptozotocin (STZ)-induced diabetic rats. Mol Cell Endocrinol.

[b28] Iwadate Y (2008). Clinical proteomics in cancer research-promises and limitations of current two-dimensional gel electrophoresis. Curr Med Chem.

[b29] Olsen JV, Ong SE, Mann M (2004). Trypsin cleaves exclusively C-terminal to arginine and lysine residues. Mol Cell Proteomics.

[b30] Eng JK, McCormack AL, Yates JR (1994). An approach to correlate tandem mass spectral data of peptides with amino acid sequences in a protein database. J Am Soc Mass Spectrom.

[b31] Minden J (2007). Comparative proteomics and difference gel electrophoresis. Biotechniques.

[b32] Kopchick JJ, List EO, Kohn DT (2002). Perspective: proteomics–see “spots” run. Endocrinology.

[b33] Yang MH, Wang HY, Lu CY (2013). Proteomic profiling for peritoneal dialysate: differential protein expression in diabetes mellitus. Biomed Res Int.

[b34] Wu Q, Yuan H, Zhang L (2012). Recent advances on multidimensional liquid chromatography-mass spectrometry for proteomics: from qualitative to quantitative analysis–a review. Anal Chim Acta.

[b35] Crimmins DL (1994). Applications of strong cation-exchange (SCX)-HPLC in synthetic peptide analysis. Methods Mol Biol.

[b36] Nakatani N, Kozaki D, Mori M (2012). Recent progress and applications of ion-exclusion/ion-exchange chromatography for simultaneous determination of inorganic anions and cations. Anal Sci.

[b37] Thingholm TE, Jensen ON, Robinson PJ (2008). SIMAC (sequential elution from IMAC), a phosphoproteomics strategy for the rapid separation of monophosphorylated from multiply phosphorylated peptides. Mol Cell Proteomics.

[b38] López E, Wesselink JJ, López I (2011). Technical phosphoproteomic and bioinformatic tools useful in cancer research. J Clin Bioinforma.

[b39] Jensen SS, Larsen MR (2007). Evaluation of the impact of some experimental procedures on different phosphopeptide enrichment techniques. Rapid Commun Mass Spectrom.

[b40] Larsen MR, Thingholm TE, Jensen ON (2005). Highly selective enrichment of phosphorylated peptides from peptide mixtures using titanium dioxide microcolumns. Mol Cell Proteomics.

[b41] Engholm-Keller K, Birck P, Størling J (2012). TiSH–a robust and sensitive global phosphoproteomics strategy employing a combination of TiO2, SIMAC, and HILIC. J Proteomics.

[b42] Kweon HK, Håkansson K (2006). Selective zirconium dioxide-based enrichment of phosphorylated peptides for mass spectrometric analysis. Anal Chem.

[b43] Nühse TS, Stensballe A, Jensen ON (2003). Large-scale analysis of in vivo phosphorylated membrane proteins by immobilized metal ion affinity chromatography and mass spectrometry. Mol Cell Proteomics.

[b44] Gruhler A, Olsen JV, Mohammed S (2005). Quantitative phosphoproteomics applied to the yeast pheromone signaling pathway. Mol Cell Proteomics.

[b45] Li X, Gerber SA, Rudner AD (2007). Large-scale phosphorylation analysis of alpha-factor-arrested Saccharomyces cerevisiae. J Proteome Res.

[b46] Albuquerque CP, Smolka MB, Payne SH (2008). A multidimensional chromatography technology for in-depth phosphoproteome analysis. Mol Cell Proteomics.

[b47] Luo R, Zhao H (2012). Protein quantitation using iTRAQ: review on the sources of variations and analysis of nonrandom missingness. Stat Interface.

[b48] Evans C, Noirel J, Ow SY (2012). An insight into iTRAQ: where do we stand now?. Anal Bioanal Chem.

[b49] Ong SE (2012). The expanding field of SILAC. Anal Bioanal Chem.

[b50] Holman SW, Sims PF, Eyers CE (2012). The use of selected reaction monitoring in quantitative proteomics. Bioanalysis.

[b51] Law KP, Lim YP (2013). Recent advances in mass spectrometry: data independent analysis and hyper reaction monitoring. Expert Rev Proteomics.

[b52] de Hoffmann E (1996). Tandem mass spectrometry: a primer. J Mass Spectrom.

[b53] Lange V, Picotti P, Domon B (2008). Selected reaction monitoring for quantitative proteomics: a tutorial. Mol Syst Biol.

[b54] Kondrat RW, McClusky GA, Cooks RG (1978). Multiple reaction monitoring in mass spectrometry/mass spectrometry for direct analysis of complex mixtures. Anal Chem.

[b55] Peterson AC, Russell JD, Bailey DJ (2012). Parallel reaction monitoring for high resolution and high mass accuracy quantitative, targeted proteomics. Mol Cell Proteomics.

[b56] Picotti P, Aebersold R (2012). Selected reaction monitoring-based proteomics: workflows, potential, pitfalls and future directions. Nat Methods.

[b57] Wang Q, Chaerkady R, Wu J (2011). Mutant proteins as cancer-specific biomarkers. Proc Natl Acad Sci U S A.

[b58] Unwin RD, Griffiths JR, Leverentz MK (2005). Multiple reaction monitoring to identify sites of protein phosphorylation with high sensitivity. Mol Cell Proteomics.

[b59] Ashman K, Villar EL (2009). Phosphoproteomics and cancer research. Clin Transl Oncol.

[b60] Matros A, Kaspar S, Witzel K (2011). Recent progress in liquid chromatography-based separation and label-free quantitative plant proteomics. Phytochemistry.

[b61] Nahnsen S, Bielow C, Reinert K (2013). Tools for label-free peptide quantification. Mol Cell Proteomics.

[b62] Bantscheff M, Schirle M, Sweetman G (2007). Quantitative mass spectrometry in proteomics: a critical review. Anal Bioanal Chem.

[b63] Asara JM, Christofk HR, Freimark LM (2008). A label-free quantification method by MS/MS TIC compared to SILAC and spectral counting in a proteomics screen. Proteomics.

[b64] Bridges SM, Magee GB, Wang N (2007). ProtQuant: a tool for the label-free quantification of MudPIT proteomics data. BMC Bioinformatics.

[b65] Mueller LN, Brusniak MY, Mani DR (2008). An assessment of software solutions for the analysis of mass spectrometry based quantitative proteomics data. J Proteome Res.

[b66] Scholl PF (2007).

[b67] Schaab C, Geiger T, Stoehr G (2012). Analysis of high accuracy, quantitative proteomics data in the MaxQB database. Mol Cell Proteomics.

[b68] Van Riper SK, de Jong EP, Carlis JV (2013). Mass spectrometry-based proteomics: basic principles and emerging technologies and directions. Adv Exp Med Biol.

[b69] Tao D, Zhang L, Shan Y (2011). Recent advances in micro-scale and nano-scale high-performance liquid-phase chromatography for proteome research. Anal Bioanal Chem.

[b70] Bingley PJ, Williams AJ (2004). Validation of autoantibody assays in type 1 diabetes: workshop programme. Autoimmunity.

[b71] Zangar RC, Daly DS, White AM (2006). ELISA microarray technology as a high-throughput system for cancer biomarker validation. Expert Rev Proteomics.

[b72] Caron M, Choquet-Kastylevsky G, Joubert-Caron R (2007). Cancer immunomics using autoantibody signatures for biomarker discovery. Mol Cell Proteomics.

[b73] Rai AJ, Gelfand CA, Haywood BC (2005). HUPO Plasma Proteome Project specimen collection and handling: towards the standardization of parameters for plasma proteome samples. Proteomics.

[b74] Rai AJ, Vitzthum F (2006). Effects of preanalytical variables on peptide and protein measurements in human serum and plasma: implications for clinical proteomics. Expert Rev Proteomics.

[b75] Walther TC, Mann M (2010). Mass spectrometry-based proteomics in cell biology. J Cell Biol.

[b76] Budde P, Schulte I, Appel A (2005). Peptidomics biomarker discovery in mouse models of obesity and type 2 diabetes. Comb Chem High Throughput Screen.

[b77] Jürgens M, Appel A, Heine G (2005). Towards characterization of the human urinary peptidome. Comb Chem High Throughput Screen.

[b78] Tammen H, Hess R, Schulte I (2005). Prerequisites for peptidomic analysis of blood samples: II. Analysis of human plasma after oral glucose challenge – a proof of concept. Comb Chem High Throughput Screen.

[b79] Choudhary C, Mann M (2010). Decoding signalling networks by mass spectrometry-based proteomics. Nat Rev Mol Cell Biol.

[b80] Aebersold R, Mann M (2003). Mass spectrometry-based proteomics. Nature.

[b81] Perez-Riverol Y, Wang R, Hermjakob H (2014). Open source libraries and frameworks for mass spectrometry based proteomics: a developer’s perspective. Biochim Biophys Acta.

[b82] Maris M, Ferreira GB, D′hertog W (2010). High glucose induces dysfunction in insulin secretory cells by different pathways: a proteomic approach. J Proteome Res.

[b83] Kim SW, Hwang HJ, Baek YM (2009). Proteomic analysis in ob/ob mice before and after hypoglycemic polysaccharide treatments. J Microbiol Biotechnol.

[b84] Alfadda AA, Benabdelkamel H, Masood A (2013). Proteomic analysis of mature adipocytes from obese patients in relation to aging. Exp Gerontol.

[b85] Cai Q, Li B, Yu F (2013). Investigation of the protective effects of phlorizin on diabetic cardiomyopathy in db/db mice by quantitative proteomics. J Diabetes Res.

[b86] Adachi J, Kumar C, Zhang Y (2007). In-depth analysis of the adipocyte proteome by mass spectrometry and bioinformatics. Mol Cell Proteomics.

[b87] Krüger M, Kratchmarova I, Blagoev B (2008). Dissection of the insulin signaling pathway via quantitative phosphoproteomics. Proc Natl Acad Sci U S A.

[b88] Walker GE, Ricotti R, Roccio M (2014). Pediatric obesity and vitamin D deficiency: a proteomic approach identifies multimeric adiponectin as a key link between these conditions. PLoS ONE.

[b89] Abu-Farha M, Tiss A, Abubaker J (2013). Proteomics analysis of human obesity reveals the epigenetic factor HDAC4 as a potential target for obesity. PLoS ONE.

[b90] Ahmed AA, Balogun KA, Bykova NV (2014). Novel regulatory roles of omega-3 fatty acids in metabolic pathways: a proteomics approach. Nutr Metab.

[b91] Benabdelkamel H, Masood A, Almidani GM (2015). Mature adipocyte proteome reveals differentially altered protein abundances between lean, overweight and morbidly obese human subjects. Mol Cell Endocrinol.

[b92] Karthik D, Vijayakumar R, Pazhanichamy K (2014). A proteomics approach to identify the differential protein level in cardiac muscle of diabeticrat. Acta Biochim Pol.

[b93] Rosenow A, Noben JP, Bouwman FG (2013). Hypoxia-mimetic effects in the secretome of human preadipocytes and adipocytes. Biochim Biophys Acta.

[b94] Belenchia AM, Tosh AK, Hillman LS (2013). Correcting vitamin D insufficiency improves insulin sensitivity in obese adolescents: a randomized controlled trial. Am J Clin Nutr.

[b95] Lubbers ER, List EO, Jara A (2013). Adiponectin in mice with altered GH action: links to insulin sensitivity and longevity?. J Endocrinol.

[b96] Hinsby AM, Olsen JV, Mann M (2004). Tyrosine phosphoproteomics of fibroblast growth factor signaling: a role for insulin receptorsubstrate-4. J Biol Chem.

[b97] Ordovás Muñoz JM (2013). Predictors of obesity: the “power” of the omics. Nutr Hosp.

[b98] Wiesenborn DS, Menon V, Zhi X (2014). The effect of calorie restriction on insulin signaling in skeletal muscle and adipose tissue of Ames dwarf mice. Aging.

[b99] Runau F, Arshad A, Isherwood J (2015). Potential for proteomic approaches in determining efficacy biomarkers following administration of fish oils rich in Omega 3 fatty acids: application in pancreatic cancers. Nutr Clin Pract.

[b100] Martos-Moreno GA, Sackmann-Sala L, Berryman DE (2013). Anatomical heterogeneity in the proteome of human subcutaneous adipose tissue. An Pediatr.

[b101] Murri M, Insenser M, Bernal-Lopez MR (2013). Proteomic analysis of visceral adipose tissue in pre-obese patients with type 2 diabetes. Mol Cell Endocrinol.

[b102] Bencharit S, Baxter SS, Carlson J (2013). Salivary proteins associated with hyperglycemia in diabetes: a proteomic analysis. Mol BioSyst.

[b103] Martos-Moreno GÁ, Sackmann-Sala L, Barrios V (2014). Proteomic analysis allows for early detection of potential markers of metabolic impairment in very young obese children. Int J Pediatr Endocrinol.

[b104] Ding J, List EO, Okada S (2009). Perspective: proteomic approach to detect biomarkers of human growth hormone. Growth Horm IGF Res.

[b105] List EO, Berryman DE, Palmer AJ (2007). Analysis of mouse skin reveals proteins that are altered in a diet-induced diabetic state: a new method for detection of type 2 diabetes. Proteomics.

[b106] Ding J, Okada S, Jørgensen JO (2011). Novel serum protein biomarkers indicative of growth hormone doping in healthy human subjects. Proteomics.

[b107] López Villar E, Wu D, Cho WC (2014). Proteomics-based discovery of biomarkers for paediatric acute lymphoblastic leukaemia: challenges and opportunities. J Cell Mol Med.

[b108] López Villar E, Wang X, Madero L (2015). Application of oncoproteomics to aberrant signalling networks in changing the treatment paradigm in acute lymphoblastic leukaemia. J Cell Mol Med.

[b109] López E, Madero L, Lopez-Pascual JA http://www.crcpress.com/product/isbn/9781771880602.

[b110] Orchard S, Albar JP, Binz PA (2014). Meeting new challenges: The 2014 HUPO-PSI/COSMOS Workshop: 13-15 April 2014, Frankfurt, Germany. Proteomics.

[b111] Frantzi M, Bhat A, Latosinska A (2014). Clinical proteomic biomarkers: relevant issues on study design & technical considerations in biomarker development. Clin Transl Med.

